# Supplementary material for: Prognostic Factors in Patients with Sudden Cardiac Arrest and Acute Myocardial Infarction Undergoing Percutaneous Interventions with the LUCAS-2 System for Mechanical Cardiopulmonary Resuscitation
Source: J Clin Med. 2022 Jul 4;11(13):3872. doi: 10.3390/jcm11133872 (PMC9267592; doi:10.3390/jcm11133872)
Supplement: Supplementary file 1 [file jcm-11-03872-s001.zip › jcm-1767586-supplementary.pdf]

Table S1 (Supplementary Table S1). Patient characteristics and outcomes

Characteristic	Overall group *n* = 71 (100%)	Survivors *n* = 17 (23.9%)	Non-survivors *n* = 54 (76.1%)	*p*-value
**Age, years**	71.7 ± 12	69.7 ± 11	72.1 ± 11	0.8
**Gender, males**	44 (62%)	14 (82%)	30 (56%)	0.1
**LVEF, %**	49.8 ± 36	48.1 ± 35.2	64.5 ± 51.6	0.8
**Myocardial infarction type**				
**NSTEMI/STEMI/unknown, (N, (%))**	13,(18)/31(44)/27(38)	2(12)/2(12)/13(76)	11(20)/29(54)/14(26)	0.0007
**Onset of cardiac arrest**				
**OHCA/IHCA, (N, (%))**	31(44)/24(34)	8(47)/2(12)	23(43)/22(41)	0.1
**Initial rhythm at cardiac arrest**				
**VF/PEA/asystole/unknown, (N, (%))**	14(19,7)/33(46,5)/11(15,5)/13(18,3)	1(6)/10(59)/0(0)/6(35)	13(24)/23(43)/11(20)/7(13)	0.1
**Patient history**				
**Hypertension, (N, (%))**	30(42)	5(29)	25(46)	0.2
**Type 2 Diabetes, (N, (%))**	12(17)	2(12)	10(19)	1.0
**Previous MI, (N, (%))**	13(18)	3(18)	10(19)	1.0
**Arrhythmias*, (N, (%))**	31(44)	20(12)	11(20)	0.7
**Valvular heart disease, (N, (%))**	4(6)	1(6)	3(6)	1.0
**CKD, (N, (%))**	24(34)	20(12)	4(7)	0.6
**ICD, N, (%))**	4(6)	1(6)	3(6)	1.0
**Angiographic findings among patients**				
**Distribution of diseased vessels**				
**LM/LAD/Cx/RCA, (N, (%))**	21(30)/51(72)/35(49)/36(51)	5(29)/13(76)/9(53)/11(65)	16(30)/38(70)/26(48)/25(46)	-
**Infarct-related arteries**				
**LM/LAD/Cx/RCA, %**	17(24)/38(54)/22(31)/19(27)	4(24)/6(35)/6(35)/6(35)	13(24)/32(59)/16(30)/13(24)	-
**TIMI grade flow at baseline**				
**TIMI LM > 1, (N, (%))**	5(7)	1(6)	4(7)	1.0
**TIMI LAD > 1, (N, (%))**	11(15)	4(24)	7(13)	0.2
**TIMI Cx> 1, (N, (%))**	6(8)	2(12)	4(7)	1.0
**TIMI RCA > 1, (N, (%))**	9(13)	2(12)	7(13)	1.0
**Multi-vessel CAD, (N, (%))**	27(38)	6(35,3)	21(39)	1.0
**2 vessels, (N, (%))**	19(27)	4(23,5)	15(28)	1.0
**3 vessels, (N, (%))**	8(11)	2(11,8)	6(11)	1.0
**Procedural data of patients**				
**Volume of contrast, mL**	180 ± 86	182 ± 62	179 ± 96	0.5
**Radiation dose, mGy**	898 ± 893	647 ± 404	999 ± 1015	0.2
**Respirator, (N, (%))**	62 (87)	16 (94)	46 (85)	1.0
**Coronarography, (N, (%))**	60 (85)	14 (82)	46 (85)	0.2
**Radial/femoral/unknown PCI access, (N, (%))**	14(20)/51(72)/6(8)	3(18)/12(71)/2(11)	11(20)/39(72)/4(8)	0.8
**PCI, (N, (%))**	56(79)	11(65)	45(83)	1.0
**Balloon angioplasty, (N, (%))**	55(77)	14(82)	41(76)	0.3
**Stent, (N, (%))**	53(75)	14(82)	39(72)	0.2
**Thrombectomy, (N, (%))**	23(32)	6(35)	17(31)	0.5
**Glycoprotein IIb/IIIa inhibitors, (N, (%))**	27(38)	6(35)	21(39)	0.7
**Endocavitary electrode, (N, (%))**	23(32)	2(12)	21(39)	0.4
**IABP, (N, (%))**	15(21)	6(35)	9(17)	0.2
**Pressor amines**				
**Adrenaline, (N, (%))**	31(44)	8(47)	23(43)	0.7
**Noradrenaline. (N, (%))**	26(37)	7(41)	19(35)	1.0
**Dobutamine, (N, (%))**	19(27)	4(24)	15(28)	0.5

**Dopamine, (N, (%))**	12(17)	3(18)	9(17)	1.0

*.LVEF: left ventricular ejection fraction; NSTEMI: non-ST segment elevation myocardial infarction; STEMI: ST segment elevation myocardial infarction; OHCA: out-of-hospital cardiac arrest; INHCA: in-hospital cardiac arrest; VF: ventricular fibrillation; PEA: pulseless electrical activity; MI: myocardial infarction; CKD: chronic kidney disease; ICD: implantable cardioverter defibrillator; LM: left main coronary artery; LAD: left anterior descending coronary artery; Cx: circumflex coronary artery; RCA: right coronary artery; TIMI: thrombolysis in myocardial ischemia; CAD: coronary artery disease; PCI: percutaneous coronary intervention; IABP: intra-aortic balloon pump.

Table S2. Patient characteristics and outcomes

Selected indices	Overall group *n* = 48 (100%)	OHCA *n* = 25 (52.1%)	IHCA *n* = 23 (47.9%)	*p*-value
**Age, years**	72.6 ± 11.37	71.4 ± 11.57	74 ± 11.27	0.4
**Gender, males**	27 (56%)	18 (72%)	9 (39%)	**0.04**
**LVEF, %**	26.8 ± 15.3	27.0 ± 13.9	26.6 ± 16.7	1.0
**Myocardial infarction type**				
**NSTEMI/STEMI/unknown, (N, (%))**	12(25)/26(54)/10(21)	5(25)/16(64)/4(16)	7(30)/10(43)/6(27)	0.3
**Initial rhythm at cardiac arrest**				
**VF/PEA/asystole/unknown, (N, (%))**	13(27)/22(46)/8(17)/5(10)	6(24)/10(40)/5(20)/4(16)	7(30)/12(52)/3(13)/1(5)	0.5
**Patient history**				
**Hypertension, (N, %))**	28(58)	14(56)	14(61)	1.0
**Type 2 Diabetes, (N, %))**	12(25)	5(20)	7(30)	0.7
**Previous MI, (N, %))**	10(21)	5(20)	5(22)	1.0
**Arrhythmias[a], (N, %))**	11(23)	7(28)	4(17)	0.4
**Valvular heart disease, (N, (%))**	4(8)	3(12)	1(4)	0.6
**CKD, (N, (%))**	5(10)	2(8)	3(13)	0.7
**ICD, (N, (%))**	3(6)	1(4)	2(9)	0.6
**Angiographic findings among patients**				
**Distribution of diseased vessels**				
**LM/LAD/Cx/RCA, (N, (%))**	13(27)/36(75)/23(48)/27(56)	6(24)/16(64)/12(48)/13(52)	7(30)/20(87)/11(48)/14(61)	-
**Infarct-related arteries**				
**LM/LAD/Cx/RCA, (N, (%))**	10(21)/22(46)/10(21)/14(29)	5(25)/9(36)/5(20)/8(32)	5(22)/13(57)/5(21)/6(26)	-
**TIMI grade flow at baseline**				
**TIMI LM > 1, (N, (%))**	29(60)	14(56)	15(65)	0.1
**TIMI LAD > 1, (N, (%))**	16(33)	10(40)	6(26)	0.3
**TIMI Cx> 1, (N, (%))**	24(50)	11(44)	13(57)	0.5
**TIMI RCA > 1, (N, (%))**	19(40)	8(32)	11(48)	0.5
**Multivessel CAD, (N, (%))**	15(31)	7(28)	8(35)	0.7
**2 vessels, (N, (%))**	11(23)	6(24)	5(22)	1.0
**3 vessels, (N, (%))**	4(8)	1(4)	3(13)	0.3
**Procedural data of patients**				
**Procedural data**				
**Volume of contrast, mL**	206.7 ± 27.5	175.7 ± 86.4	250 ± 149.8	0.5
**Radiation dose, mGy**	1130.8 ± 1150.0	707.5 ± 252.3	1796 ± 1672.2	**0.03**
**Respirator, (N, (%))**	85	88	83	0.5
**Radial/femoral/unknown PCI access, (N, (%))**	10(21)/33(69)/5(10)	2(8)/19(76)/4(16)	8(35)/14(61)/1(4)	**0.047**
**PCI, (N, (%))**	45(94)	23(92)	22(96)	1.0
**Balloon angioplasty, (N, (%))**	39(81)	19(76)	20(87)	0.1
**Stent, (N, (%))**	36(75)	19(76)	17(74)	1.0
**Thrombectomy, (N, (%)**	13(27)	5(20)	8(35)	0.3
**GlycoproteinIIb/IIIa inhibitors, (N, (%))**	18(38)	9(36)	9(39)	0.8

**Endocavitary electrode, (N, (%))**	16(33)	5(20)	11(48)	0.1
**IABP, (N, (%))**	9(19)	5(20)	4(17)	1.0
**Pressoramines**				
**Adrenaline, (N, (%))**	21(44)	9(36)	12(52)	0.5
**Noradrenaline. (N, (%))**	22(46)	12(48)	10(43)	0.3
**Dobutamine, (N, (%))**	18(38)	9(36)	9(39)	0.7
**Dopamine, (N, (%))**	13(27)	10(40)	3(13)	**0.01**

*.Data are shown as mean ± standard deviation (SD) or proportions (%). *P*-values below 0.05 were marked in bold.

[a] Arrythmias include: atrial fibrillation, 3rd degree cardiac block and left bundle branch block.

LVEF: left ventricular ejection fraction; NSTEMI: non-ST segment elevation myocardial infarction; STEMI: ST segment elevation myocardial infarction; OHCA: out-of-hospital cardiac arrest; INHCA: in-hospital cardiac arrest; VF: ventricular fibrillation; PEA: pulseless electrical activity; MI: myocardial infarction; CKD: chronic kidney disease; ICD: implantable cardioverter defibrillator; LM: left main coronary artery; LAD: left anterior descending coronary artery; Cx: circumflex coronary artery; RCA: right coronary artery; TIMI: thrombolysis in myocardial ischemia; CAD: coronary artery disease; PCI: percutaneous coronary intervention; IABP: intra-aortic balloon pump.

Table S3. Laboratory data

Selected indices	Overall group *n* = 48 (100%)	OHCA *n* = 25 (52.1%)	IHCA *n* = 23 (47.9%)	*p*-value
**aPTT, s**	83.9 ± 70.3	85.8 ± 68.3	81.3 ± 75.4	0.7
**INR**	1.5 ± 1.1	1.6 ± 1.3	1.4 ± 0.7	1.0
**Prothrombin time, s**	17.3 ± 11.8	18.3 ± 14.5	16.1 ± 7.5	0.9
**Hemoglobin, g/dL**	12.4 ± 2.3	12.6 ± 2.3	12.1 ± 2.3	0.6
**Erythrocytes, x10^6/µL**	4.1 ± 0.7	4.0 ± 0.7	4.2 ± 0.6	0.5
**Platelets, x10^3/µL**	193.7 ± 75.6	168.0 ± 65.5	223.7 ± 77.1	**0.02**
**Creatinine, mg/dL**	1.8 ± 1.2	1.79 ± 1.1	1.82 ± 1.4	0.4
**eGFR, mL/min/1.73 m^2**	47.0 ± 23.4	45.5 ± 16.8	48.6 ± 28.9	0.7
**Sodium, mmol/L**	138.6 ± 4.9	139.2 ± 5.6	138 ± 4.2	0.3
**Potassium, mmol/L**	4.5 ± 0.9	4.3 ± 0.8	4.7 ± 1.0	0.2

*aPTT: activated partial thromboplastin time; INR: international normalized ratio; eGFR: estimated glomerular filtration rate.

Table S4. Univariate logistic regression analysis of survival predictors with laboratory parameters as dichotomous variables

Predictor	Odds ratio	95% Confidence Interval	*p*-value
**Gender, male vs. female**	1.65	0.70-3.89	0.3
**Prothrombin time >15 s**	1.94	0.60-6.28	0.3
**Hemoglobin < 10 g/dL**	1.53	0.41-5.73	0.5
**Platelets < 100x103/µL**	2.24	0.57-8.76	0.3
**Creatinine > 1.2 mg/dL**	1.50	0.51-4.42	0.5
**eGFR < 60 mL/min/1.73 m2**	1.65	0.56-4.85	0.4
**Sodium < 135 mmol/L**	0.92	0.19-4.37	0.9
**Potassium > 5.0 mmol/La**	4.61	1.41-15.05	**0.01**
**Glucose > 180 mg/dL**	0.55	0.12-2.60	0.5
**CRP > 5 mg/dL**	1.41	0.38-5.24	0.6
**pH < 7.35**	1.04	0.30-3.58	1.0
**pH < 7.15**	0.75	0.28-1.99	0.6
**pCO2 > 45 mmHg**	1.33	0.50-3.53	0.6

[a]Age- and sex-adjusted ORs and *p*-values.
